# Applications of Probiotics and Their Potential Health Benefits

**DOI:** 10.3390/ijms242115915

**Published:** 2023-11-02

**Authors:** Virginia Fuochi, Pio Maria Furneri

**Affiliations:** Department of Biomedical and Biotechnological Sciences (BIOMETEC), University of Catania, 95124 Catania, Italy; furneri@unict.it

Probiotics have garnered significant attention in recent years due to their potential health benefits and their role in promoting a balanced gut microbiome. This topic was aimed at delving into the applications of probiotics and their wide-ranging impacts on human health. The 20th century witnessed a significant shift in probiotic research, starting with the groundbreaking work of the scientist Elie Metchnikoff. He postulated that the consumption of lactic acid bacteria, commonly found in fermented dairy products, could confer health benefits by modulating the gut microbiota. His pioneering ideas paved the way for further scientific inquiries into the world of probiotics.

Recently, innovative methods have been developed for the discovery of strains that could be beneficial for both humans and livestock animals [1,2,3]. Phenotypic tests could be employed to assess the necessary characteristics for strains to be considered probiotics, such as resistance to bile salts, cytoprotective effects against oxidative stress, and inhibition of pathogens [4,5,6,7]. Moreover, it appears that artificial intelligence algorithms could identify new probiotics and distinguish them from pathogens in the human gut by determining the informational content within tRNA sequences as key genomic features for probiotic characterization [8]. Furthermore, transcriptomic analysis has proven to be valuable in assessing the potential antimicrobial mechanisms exhibited by certain probiotic strains like *Lactobacillus rhamnosus* SCB0119 [9].

One of the most intriguing aspects of probiotics is their potential to modulate the immune system. Research suggests that certain strains of probiotics could enhance both innate and adaptive immune responses. This modulation could have far-reaching implications for conditions ranging from allergies to autoimmune disorders, offering a promising avenue for therapeutic intervention. For instance, treatment with *L. reuteri* could regulate the intestinal microbial composition and enhance tryptophan metabolism, leading to the production of aryl hydrocarbon receptor ligands, including indole lactic acid and indole-propionic acid. These ligands activate AHR signaling, effectively reducing the aberrant Th2-type response, and prove to be an effective alternative for alleviating atopic dermatitis [10]. Further, the administration of a heat-killed mixture of *Lactococcus lactis* subsp. *cremoris* and *L. paracasei* subsp. *paracasei* demonstrated the modulation of immune T cell balance and the suppression of IgE production in mice with house dust mite extract-induced atopic dermatitis, thus reducing the associated symptoms [11].

Several studies have demonstrated that not only bacterial cells themselves, but also their supernatant products, induce immunomodulatory activity by stimulating phagocytosis in macrophagic cells, thereby enhancing the expression of immunomodulators such as NO, TNF-alpha, IL-6, iNOS, and COX-2 [12]. Furthermore, it has been demonstrated that certain probiotic strains are able to exert their immunomodulatory properties in mucosal sites, both when alive and when inactivated. For instance, it appears that the mbf protein is not implicated in the immunobiotic effects induced by these strains, providing equal protection against inflammatory damage [13].

Probiotics have shown remarkable promise in managing various gastrointestinal disorders. Conditions such as irritable bowel syndrome, inflammatory bowel disease, gastric and colorectal cancers, and antibiotic-associated diarrhea have all been the subjects of extensive probiotic research [14]. Studies indicate that specific strains of probiotics could alleviate symptoms, reduce inflammation, and restore gut homeostasis in individuals suffering from these conditions. For instance, heat-killed *B. bifidum* MG731, *L. reuteri* MG5346, and *L. rhamnosus* MG5200 induced greater apoptosis in human gastric cancer MKN1 cells. Oral administration of a single dose of this mixture significantly delayed tumor growth [15]. Moreover, strains such as *Bifidobacterium longum*, *Lactobacillus plantarum* and *Pediococcus acidilactici* have shown to alleviate ETEC-induced diarrhea by regulating the immune response, rebalancing intestinal microbiota, and improving carbohydrate metabolism [16]. Therefore, probiotics have demonstrated a noteworthy anti-inflammatory effect, showcasing their potential as a valuable intervention in managing inflammatory conditions [17].

The role of probiotics in metabolic health has garnered increasing attention, particularly in the context of obesity and hypercholesterolemia. For instance, oral administration of recombinant probiotics expressing lactoferrin could improve diet-induced lipid accumulation and inflammation in non-alcoholic fatty liver disease [18]. Moreover, emerging evidence suggests that certain probiotic strains may influence metabolic pathways, leading to promotion of the intestinal transformation of ellagic acid, which leads to the upregulation of liver bile synthesis, thus preventing hypercholesterolemia [19]. These findings hold significant implications for the prevention and management of metabolic disorders.

The gut–brain axis, a bidirectional communication network between the gastrointestinal tract and the central nervous system, has emerged as a pivotal area of research. Probiotics, by modulating the gut microbiota, may have the potential to impact mental health conditions such as anxiety, depression, and even cognitive functioning [20]. This exciting avenue of research underscores the holistic nature of probiotic health benefits.

The application of probiotics in pulmonary medicine represents a particularly promising frontier. Studies have demonstrated the potential of probiotics for preventing and treating conditions like COVID-19 [21]. These findings open up new avenues for improving health-stimulating pulmonary immunity through the intestine–lung axis.

While the potential benefits of probiotics are substantial, several challenges and considerations also warrant attention. These include strain-specific effects, individual variabilities in microbial composition, and the need for personalized probiotic interventions. Additionally, regulatory standards for probiotic products vary globally, underscoring the importance of robust research and quality control measures. Furthermore, the advancement of probiotics as potent treatments for human health problems relies heavily on surmounting the technological challenges to production. By prioritizing cellular vitality, component integrity, and host interactions, and through the careful selection of strains, we can pave the way for probiotics to reach their full potential in promoting human health and well-being [22]. This endeavor demands collaborative efforts from researchers, technologists, and healthcare professionals to drive innovation and ensure a healthier future.

In conclusion, nowadays, the study of probiotics is approached through the One Health perspective, acknowledging the intricate interplay between human, animal, and environmental health [2,23,24]. This underscores the imperative for interdisciplinary collaboration in tackling global health issues, recognizing that the welfare of humans, animals, and ecosystems is intimately intertwined.

## References

[1] Du L., Chen W., Wang J., Huang L., Zheng Q., Chen J., Wang L., Cai C., Zhang X., Wang L. (2023). Beneficial Effects of Bacillus amyloliquefaciens D1 Soy Milk Supplementation on Serum Biochemical Indexes and Intestinal Health of Bearded Chickens. Microorganisms.

[2] Zhu Q., Azad M.A.K., Dong H., Li C., Li R., Cheng Y., Liu Y., Yin Y., Kong X. (2023). Sow-Offspring Diets Supplemented with Probiotics and Synbiotics Are Associated with Offspring’s Growth Performance and Meat Quality. Int. J. Mol. Sci..

[3] Ma K., Chen W., Lin X.Q., Liu Z.Z., Wang T., Zhang J.B., Zhang J.G., Zhou C.K., Gao Y., Du C.T. (2023). Culturing the Chicken Intestinal Microbiota and Potential Application as Probiotics Development. Int. J. Mol. Sci..

[4] Puvanasundram P., Chong C.M., Sabri S., Yusoff M.S.M., Lim K.C., Karim M. (2022). Efficacy of Single and Multi-Strain Probiotics on In Vitro Strain Compatibility, Pathogen Inhibition, Biofilm Formation Capability, and Stress Tolerance. Biology.

[5] Tian X.F., Zhu X., Wang M., Guo T., Kong J. (2023). Stimulation of Heme-Dependent Catalase Enhanced the Cytoprotective Effect of Lactobacillus plantarum against Oxidative Stress. Appl. Microbiol..

[6] Jiang Z., Su W., Yang M., Li W., Gong T., Zhang Y., Wen C., Wang X., Wang Y., Jin M. (2022). Screening of Bacteria Inhibiting Clostridium perfringens and Assessment of Their Beneficial Effects In Vitro and In Vivo with Whole Genome Sequencing Analysis. Microorganisms.

[7] Stivala A., Carota G., Fuochi V., Furneri P.M. (2021). Lactobacillus rhamnosus AD3 as a Promising Alternative for Probiotic Products. Biomolecules.

[8] Bergamini C.M., Bianchi N., Giaccone V., Catellani P., Alberghini L., Stella A., Biffani S., Yaddehige S.K., Bobbo T., Taccioli C. (2022). Machine Learning Algorithms Highlight tRNA Information Content and Chargaff’s Second Parity Rule Score as Important Features in Discriminating Probiotics from Non-Probiotics. Biology.

[9] Peng H., Zhou G., Yang X.M., Chen G.J., Chen H.B., Liao Z.L., Zhong Q.P., Wang L., Fang X., Wang J. (2022). Transcriptomic Analysis Revealed Antimicrobial Mechanisms of Lactobacillus rhamnosus SCB0119 against *Escherichia coli* and *Staphylococcus aureus*. Int. J. Mol. Sci..

[10] Fang Z., Pan T., Wang H., Zhu J., Zhang H., Zhao J., Chen W., Lu W. (2022). Limosilactobacillus reuteri Attenuates Atopic Dermatitis via Changes in Gut Bacteria and Indole Derivatives from Tryptophan Metabolism. Int. J. Mol. Sci..

[11] Chen H.Y., Chen Y.T., Li K.Y., Huang H.W., Lin Y.C., Chen M.J. (2022). A Heat-Killed Probiotic Mixture Regulates Immune T Cells Balance and IgE Production in House Dust Mite Extraction-Induced Atopic Dermatitis Mice. Microorganisms.

[12] Lee J., Kim S., Kang C.H. (2022). Immunostimulatory Activity of Lactic Acid Bacteria Cell-Free Supernatants through the Activation of NF-kappaB and MAPK Signaling Pathways in RAW 264.7 Cells. Microorganisms.

[13] Tomotsune K., Raya Tonetti F., Mizuno H., Elean M., Fukuyama K., Zhou B., Ikeda-Ohtsubo W., Nishiyama K., Yamamura A., Karasawa H. (2022). The Mucus Binding Factor Is Not Necessary for *Lacticaseibacillus rhamnosus* CRL1505 to Exert Its Immunomodulatory Activities in Local and Distal Mucosal Sites. Int. J. Mol. Sci..

[14] Fuochi V., Spampinato M., Distefano A., Palmigiano A., Garozzo D., Zagni C., Rescifina A., Li Volti G., Furneri P.M. (2023). Soluble peptidoglycan fragments produced by Limosilactobacillus fermentum with antiproliferative activity are suitable for potential therapeutic development: A preliminary report. Front. Mol. Biosci.

[15] Kim S., Lee H.H., Choi W., Kang C.H., Kim G.H., Cho H. (2022). Anti-Tumor Effect of Heat-Killed Bifidobacterium bifidum on Human Gastric Cancer through Akt-p53-Dependent Mitochondrial Apoptosis in Xenograft Models. Int. J. Mol. Sci..

[16] Li W., Kai L., Jiang Z., He H., Yang M., Su W., Wang Y., Jin M., Lu Z. (2022). Bifidobacterium longum, *Lactobacillus plantarum* and Pediococcus acidilactici Reversed ETEC-Inducing Intestinal Inflammation in Mice. Microorganisms.

[17] Foey A., Habil N., Strachan A., Beal J. (2022). Lacticaseibacillus casei Strain Shirota Modulates Macrophage-Intestinal Epithelial Cell Co-Culture Barrier Integrity, Bacterial Sensing and Inflammatory Cytokines. Microorganisms.

[18] Liu Z.S., Li P.L., Ku Y.W., Chen P.W. (2022). Oral Administration of Recombinant Lactoferrin-Expressing Probiotics Ameliorates Diet-Induced Lipid Accumulation and Inflammation in Non-Alcoholic Fatty Liver Disease in Mice. Microorganisms.

[19] Jin L., Dang H., Wu J., Yuan L., Chen X., Yao J. (2023). Supplementation of Weizmannia coagulans BC2000 and Ellagic Acid Inhibits High-Fat-Induced Hypercholesterolemia by Promoting Liver Primary Bile Acid Biosynthesis and Intestinal Cholesterol Excretion in Mice. Microorganisms.

[20] Laudani S., Torrisi S.A., Alboni S., Bastiaanssen T.F.S., Benatti C., Rivi V., Moloney R.D., Fuochi V., Furneri P.M., Drago F. (2023). Gut microbiota alterations promote traumatic stress susceptibility associated with p-cresol-induced dopaminergic dysfunctions. Brain. Behav. Immun..

[21] Valdebenito-Navarrete H., Fuentes-Barrera V., Smith C.T., Salas-Burgos A., Zuniga F.A., Gomez L.A., Garcia-Cancino A. (2023). Can Probiotics, Particularly Limosilactobacillus fermentum UCO-979C and *Lacticaseibacillus rhamnosus* UCO-25A, Be Preventive Alternatives against SARS-CoV-2?. Biology.

[22] Mendonca A.A., Pinto-Neto W.P., da Paixao G.A., Santos D.D.S., De Morais M.A., De Souza R.B. (2022). Journey of the Probiotic Bacteria: Survival of the Fittest. Microorganisms.

[23] Moravkova M., Kostovova I., Kavanova K., Pechar R., Stanek S., Brychta A., Zeman M., Kubasova T. (2022). Antibiotic Susceptibility, Resistance Gene Determinants and Corresponding Genomic Regions in *Lactobacillus amylovorus* Isolates Derived from Wild Boars and Domestic Pigs. Microorganisms.

[24] Maftei N.M., Iancu A.V., Elisei A.M., Gurau T.V., Ramos-Villarroel A.Y., Lisa E.L. (2023). Functional Characterization of Fermented Beverages Based on Soy Milk and Sea Buckthorn Powder. Microorganisms.

